# JOURNEYING BEYOND CARE: A CAREGIVER’S PERSPECTIVES ON AGING AND HEALTH PLANNING

**DOI:** 10.1093/geroni/igae098.0863

**Published:** 2024-12-31

**Authors:** Shelbie Turner, Roberta Cruz, Carolyn Malone, Gary Epstein-Lubow, Monica Moreno, Emily Mroz

**Affiliations:** Weill Cornell Medicine, New York, New York, United States; National Alzheimer’s Project ACT (NAPA) Advisory Council, San Diego, California, United States; Brown University, Providence, Rhode Island, United States; Education Development Center, Waltham, Massachusetts, United States; Alzheimer’s Association, Chicago, Illinois, United States; Emory University, Decatur, Georgia, United States

## Abstract

Emerging research suggests that caring for an older family member influences how caregivers think about, anticipate, and plan for their own aging and health changes. In 2017, nearly all (91%) caregivers participating in the University of Michigan’s National Poll on Healthy Aging reported that being a caregiver made them think about their own needs for caregiving in the future. But the pathways between caregiving, planning for health changes, and (re)defining aging self-perceptions are complex. Research on these pathways benefits from collaborative partnership with individuals with lived experiences. In this special session, we will hold a conversation with Roberta Cruz, a caregiver for her mother who has been living with dementia since 2014. Roberta is a member of the National Institute on Aging’s IMPACT Collaboratory Lived Experience Panel and the National Alzheimer’s Project ACT (NAPA) Advisory Council. She is excited to bring her voice to the Gerontological Society of America for the first time. During this conversation, to set the stage, we will present descriptive findings on caregivers’ perceptions of their future from waves of the Michigan National Poll on Healthy Aging. We will hear Roberta’s perspectives on how caregiving can evoke positive and negative views on one’s own aging and health changes, and the extent to which caregiving can be reasonably expected to guide one’s own preparedness for aging and health changes. Roberta will reflect on the presentations in this symposium and offer insights for building successful partnerships with individuals with lived experience as this area of research expands.

